# Morphological Variation of the Scorpionfly *Panorpa obtusa* Cheng (Mecoptera: Panorpidae) with a New Synonym

**DOI:** 10.1371/journal.pone.0108545

**Published:** 2014-09-24

**Authors:** Na Ma, Guilin Hu, Junxia Zhang, Baozhen Hua

**Affiliations:** State Key Laboratory of Crop Stress Biology for Arid Areas, Key Laboratory of Plant Protection Resources and Pest Management, Ministry of Education, Entomological Museum, Northwest A & F University, Yangling, Shaanxi, China; University of Tours, France

## Abstract

**Background:**

The overabundance of synonyms is an unavoidable by-product of taxonomic practice in insects. How to reduce or even eliminate synonymy has long been a great challenge for insect taxonomists. The scorpionflies *Panorpa obtusa* Cheng, 1949 and *Panorpa leei* Cheng, 1949 (Insecta: Mecoptera: Panorpidae) were originally described from Taibaishan in the Qinling Mountains with identical collection data and both are based on a single gender, the former on a male and the latter on two females. However, whether *P. leei* is conspecific with *P. obtusa* or a good species remains an unsolved problem.

**Results:**

On the basis of intensive morphological comparison of 93 males and 53 females of scorpionflies collected from the type locality using light and scanning electron microscopy, we found *P. obtusa* has considerable morphological variation (especially the wing markings and genitalia in both male and female), and *Panorpa leei* is totally comprised of one of the morphs of *P. obtusa*.

**Conclusions:**

In combination with identical type localities and overlapping morphological variation, *P. leei* Cheng is proposed as a junior subjective synonym of *P. obtusa* Cheng. To avoid synonyms, taxonomists should pay more attention to individual variation and base decisions on a series of specimens to describe new species.

## Introduction

As a fundamental science of classifying and naming living organisms, taxonomy is of vital importance in biology, especially in the face of the current global biodiversity crisis [1], [2]. Occurrence of synonyms is an annoying problem and difficult to avoid in taxonomic practice of insects over the past 250 years and imperils the estimation of alpha-biodiversity as well as any science discipline that depends on such estimates, such as community ecology, conservation biology, and biogeography [3], [4].

Panorpidae currently consists of more than 420 described extant species and above 90% species have been assigned to *Panorpa* Linnaeus, 1758 and *Neopanorpa* Weele, 1909 [5]. The adults are relatively weak flyers and are largely confined to fragmented habitats by strict ecological requirements, therefore the gene exchange between allopatric conspecific populations is often limited to a great extent, leading to complicated intraspecific morphological variation, especially the body color, wing markings, and genitalia [6], [7]. However, a number of species of Panorpidae are described on the basis of only a few specimens, even one sex or a single specimen, with the possible intraspecific morphological variation being largely or totally ignored. For example, eight species of *Panorpa* and seven species of *Neopanorpa* were described on the basis of male holotypes alone, and eleven species of *Panorpa* and ten species of *Neopanorpa* were described on the basis of only females for Chinese Panorpidae, roughly accounting for half the described species [8]. *Panorpa obtusa* Cheng, 1949 and *Panorpa leei* Cheng, 1949 are two such examples.

*Panorpa obtusa* Cheng, 1949 was described on the basis of a single male holotype, which was collected from Taibaishan in the Qinling Mountains, central Shaanxi Province, China on 14 July 1943 by Chuan Lung Lee. *Panorpa leei* Cheng, 1949 was described in the same paper based on two female type specimens with identical collecting data to *P. obtusa*, including the collection date, type locality, and collector [9]. Later, these two names were mentioned again in the revision of the Chinese Mecoptera without any additional information [8].

Chou et al. (1981) additionally described the female specimens of *P. obtusa* from Taibaishan, but ironically did not record the occurrence of *P. leei* from the same locality [10]. Instead, they recorded male specimens of *P. leei* from Huanglongshan, a locality about 350 km northeast of the type locality of the species. This raises a currently unanswered question: is *P. leei* conspecific with *P. obtusa* or a good species?

In this study, we investigated the morphological variation of both sexes of *P. obtusa* using light and scanning electron microscopy, expecting to clarify the status of *Panorpa leei* Cheng, 1949 and to elucidate if *Panorpa leei* Cheng, 1949 is a junior subjective synonym of *Panorpa obtusa* Cheng, 1949.

### Ethics Statement

No specific permits were required for the described field studies: a) no specific permissions were required for these locations/activities; b) locations were not privately-owned or protected; and c) the field studies did not involve endangered or protected species.

## Materials and Methods

A large number of adults of *Panorpa obtusa* were captured at Taibaishan in the Qinling Mountains, the type locality of *P. obtusa* and *P. leei*, from 1997 to 2013. To study the morphological variation, specimens preserved in 70% ethanol were randomly selected, including 93 males and 53 females. Specimens were observed and dissected under a Nikon SMZ1500 Stereoscopic Zoom Microscope. Male and female genitalia dissected were macerated in cold 4% NaOH for several hours and rinsed in distilled water. Photographs were taken with a QImaging Retiga 2000R Fast 1394 Digital CCD camera attached to the Nikon SMZ1500 microscope. The pictures were further treated with Syncroscopy Auto-Montage software. The wing and genital components were measured using a calibrated ocular micrometer under a Nikon SMZ1500 microscope.

The mean and standard deviation of structure length or width were calculated mainly based on 93 males and 53 females using Microsoft Excel 2007. The frequency of each morph type was also calculated in different structures, including the male hypovalve, paramere, and female subgenital plate respectively based on 85, 90, and 33 specimens. The voucher specimens are preserved in 70% ethanol at the Entomological Museum, Northwest A&F University, China (NWAU).

For scanning electron microscopy (SEM), live specimens were fixed in Carnoy’s solution (95% ethanol: glacial acetic acid = 3∶1, v/v) for 12 hours before being stored in 70% ethanol. The genitalia were dissected in 70% ethanol under a Motic K-400L microscope, and were dehydrated in a graded ethanol series after ultrasonic cleaning for 2 minutes. Then the samples were dried with liquid carbon dioxide in a critical-point dryer, sputter-coated with gold, and examined in a JEOL JSM-6360LV scanning electron microscope (JEOL, Tokyo, Japan) at 15 kV.

## Results

### *Panorpa obtusa* Cheng, 1949

*Panorpa obtusa* Cheng, 1949∶ 142, figs. 2, 25, 27, 30; Cheng, 1957∶ 23, figs. 28, 34, 37, 272; Chou et al., 1981∶ 14, figs. 53–56.

*Panorpa leei* Cheng, 1949∶ 147, figs. 17, 18, 54; Cheng, 1957∶ 53, fig. 125, 127, 275; Chou et al., 1981∶ 11, figs. 40–43. **syn. nov.**

#### Males

**Figure 1:**
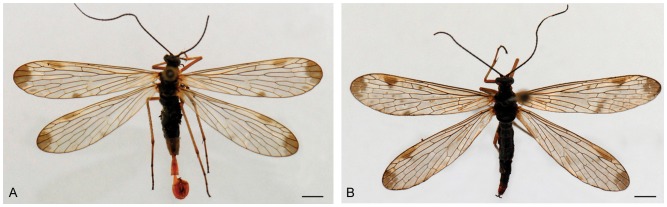
*Panorpa obtusa*, adults in dorsal view. (**A**) male. (**B**) Female. Scale bars = 2 mm.

**Figure 2:**
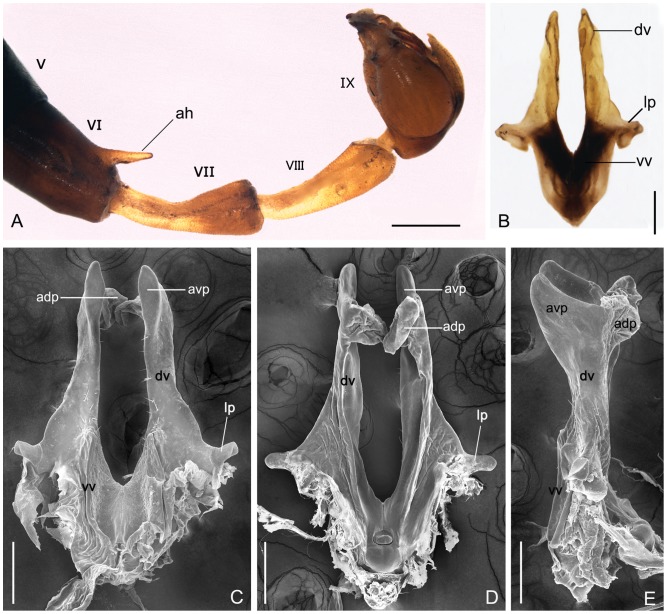
Male abdomen and aedeagus of *P. obtusa.* (**A**) Light micrograph of the abdomen in lateral view. (**B**) Light micrograph of the aedeagus, ventral view. (**C**)–(**E**) SEM of the aedeagus in ventral, dorsal, and lateral views. The apicodorsal processes are artificially malformed during the process of sample preparation. adp, apicodorsal process; ah, anal horn; avp, apicoventral process; dv, dorsal valve; lp, lateral process; V–IX, the abdominal segment V–IX; vv, ventral valve. Scale bars: (**A**) = 0.1 mm; (**B**)–(**E**) = 0.2 mm.

Medium-sized (Fig. 1A). Vertex entirely black; ocellar triangle and frons black; rostrum yellowish brown to reddish brown, with a weakly defined grayish stripe on each side; thorax reddish brown laterally, entirely black dorsally; the first six abdominal segments black dorsally and ventrally, terminal abdominal segments reddish brown, the hind border of the third tergite prolonged into a small semicircular process (the notal organ), the sixth abdominal tergum produced backward into a single median anal horn, reddish brown in color (Fig. 2A). Forewing length 12.9±0.5 mm, width 3.5±0.16 mm. Wings mostly dusky hyaline, without markings or with a slight indication of grayish brown at the apex; the wing apex obtuse and broad. Hindwing length 12.0±0.5 mm, width 3.40±0.15 mm, similar to the forewing.

Genital bulb rounded, gonocoxite 1.77±0.08 mm long. Gonostylus 1.04±0.03 mm long, much shorter than gonocoxite; the outer margin smoothly curved, with a greatly reduced median tooth and a large basal concave area on the inner margin. Hypandrium (9th sternum) 1.30±0.09 mm long, without a distinct stalk, paired elongate hypovalves bearing long bristles along the inner margin and divergent from the base, nearly reaching the base of gonostylus. Epandrium (9th tergum) slightly tapering toward the apex with wide V-shaped or deep U-shaped emargination. Parameres long and curved, extending mostly beyond the tips of gonocoxites and reaching the middle part of the gonostylus; each paramere consisting of a slender basal stalk, a broad middle plate, and a hairy distal half; the basal stalk weakly chitinized, bearing a short process at the mid-lateral part; the middle plate varying in shape among individuals; and the distal half furnished with a series of long spines along the inner margins. Aedeagus consisting of a pair of ventral valves and a pair of dorsal valves; the ventral valves membranous and undeveloped; the dorsal valves developed and bearing a small digitiform lateral process at the middle part, a broad apicoventral process and a flat membranous apicodorsal process shaped like a fishtail in lateral view; a button-shaped structure located on the basodorsal part (Figs. 2B−D).

#### Females

Vertex black; rostrum reddish brown, with a short and deep brown stripe on each side of its upper portion; thorax black dorsally, yellowish brown laterally; the first six abdominal segments black dorsally and ventrally, the 7th to 9th abdominal segments short and reddish brown. Forewing length 13.1±0.5 mm, width 3.7±0.2 mm. Wing markings indistinct or distinct, varying greatly. Hindwing length 11.9±0.4 mm, width 3.6±0.2 mm, similar to the forewing (Fig. 1B).

**Figure 3:**
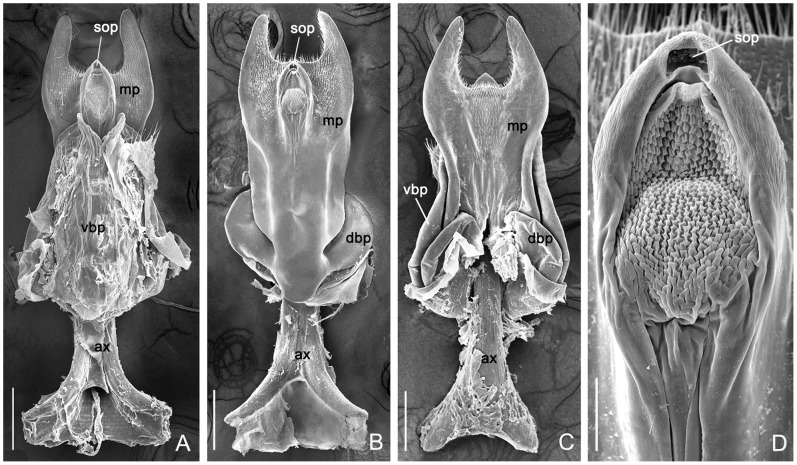
SEM of the female genital plate of *P. obtusa.* (**A**) Ventral view. (**B**) Ventral view with the ventral basal plate removed. (**C**) Dorsal view. (**D**) Magnification of the distal part of axis in B, with the sculpturing part partly artificial deformed. ax, axis; dbp, dorsal basal plate; mp, main plate; spo, orifice of spermathecal duct; vbp, ventral basal plate. Scale bars: (**A**)–(**C**) = 200 µm; (D) = 40 µm.

Subgenital plate small, narrowed posteriorly, apex emarginated. Genital plate complicated, consisting of a main plate, an axis, a pair of posterior arms, and two pairs of basal plates. The main plate abruptly narrow at the base, with paired ventral and dorsal basal plates curving dorsad at the lateral margins; ventral basal plates elongate and fused basally, only bifurcated at the apical part; dorsal basal plates relatively small; a pair of posterior arms acute and U-shaped; axis bearing a regular sculptured oval region posteriorly and the copulatory pore at its terminal end; the anterior part of the axis bifurcated, each side emarginate distally on the lateral side; the axis extending beyond the plate nearly half its length; numerous microtrichia distributed densely on the posterior arms ventrally and on the posterior part of main plate both ventrally and dorsally (Fig. 3).

#### Variation

*P. obtusa* exhibits great morphological variation both in males and females, especially on the wing pattern and genitalia. The morphological variation of individuals is continuous, lacking remarkable correlation between each two morph types.

#### Variation of the male wings

**Figure 4:**
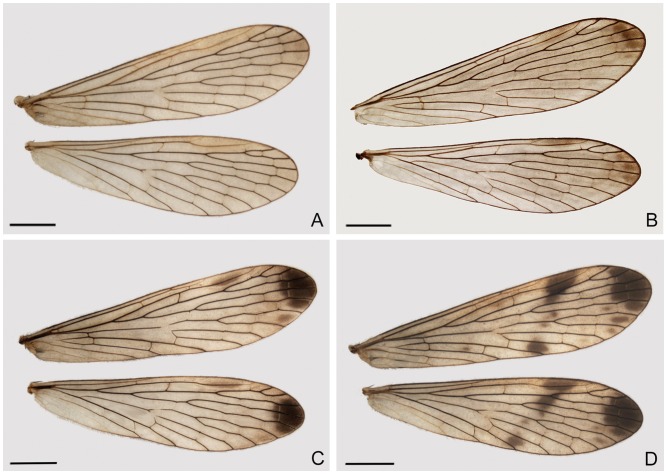
Variation of male wing markings of *P. obtusa*. Scale bars = 2 mm.

Four distinct types of wing patterns can be distinguished. Type I (47.31%) has dusky hyaline wings, without markings (Fig. 4A). Type II (25.81%) has wings that lack markings except for an indistinct grayish brown apical band and pterostigma (Fig. 4B). Wings of type III (23.66%) bear a distinct dark brown apical band and prominent pterostigma (Fig. 4C). Type IV wings (3.23%) have a distinct apical band and a dark brown pterostigmal band, which is broken and comprises only a basal branch (Fig. 4D). Among these variation types, nearly half of wings (type I) are dusky hyaline, without markings. Type IV is considerably rare.

#### Variation of the male genitalia

**Figure 5:**
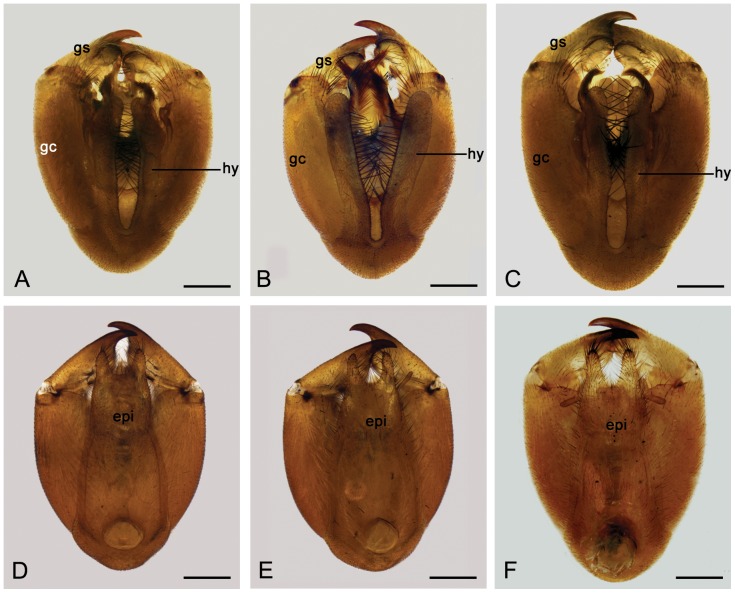
Variation of male genitalia of *P. obtusa*. (**A**)–(**C**) Ventral view. (**D**)–(**F**) Dorsal view. epi, epandrium; gc, gonocoxite; gs, gonostylus; hy, hypovalve. Scale bars = 0.4 mm.

**Figure 6:**
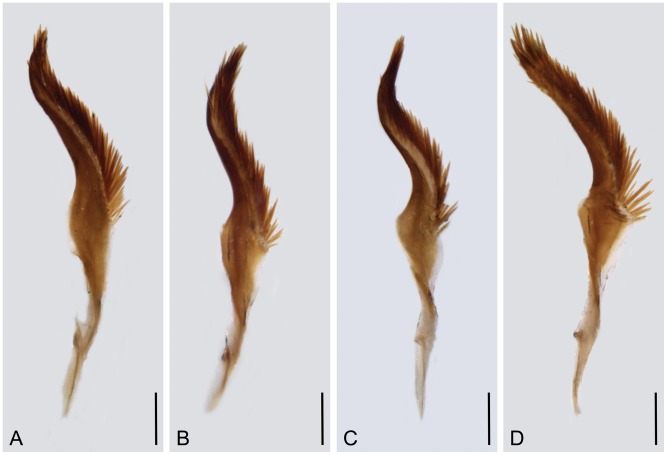
Variation of male paramere of *P. obtusa*. Scale bars = 0.2 mm.

The male genitalia vary distinctly in size. For example, the length of gonocoxite varies from 1.56 to 1.86 mm and gonostylus from 0.96 to 1.13 mm. The hypovalves vary from 1.10 to 1.46 mm in length, being divergent basally and arranged in a broad V-shape (type I, 54.12%, Fig. 5A), narrow U-shape (type II, 42.35%, Fig. 5B), or broad U-shape basally but abruptly narrower at middle (type III, 3.53%, Fig. 5C). Most hypovalves are 1.38–1.46 mm long and arranged in a broad V-shape basally (type I). The epandrium varies mainly in its apical emargination as three distinct types: inverted trapezoid-shaped (type I, 63.44%, Fig. 5D), broad U-shaped (type II, 36.56%, Fig. 5E), and deep V-shaped (type III, 3.23%, Fig. 5F). The paramere is variable in the curvature degree of the outer and inner margins and can be categorized into four main types. Type I (22.22%) is characterized by the outer and inner margins curving smoothly (Fig. 6A). In type II (24.44%) the outer margin curves smoothly, but the inner margin curves sharply in the middle (Fig. 6B). In type III (31.11%) the outer margin curves sharply in the middle, but the inner margin curves smoothly (Fig. 6C). Type IV (22.22%) of paramere is sharply curved in the middle of the outer and inner margins (Fig. 6D). All types of parameres have similar frequency.

#### Variation of the female wings

**Figure 7:**
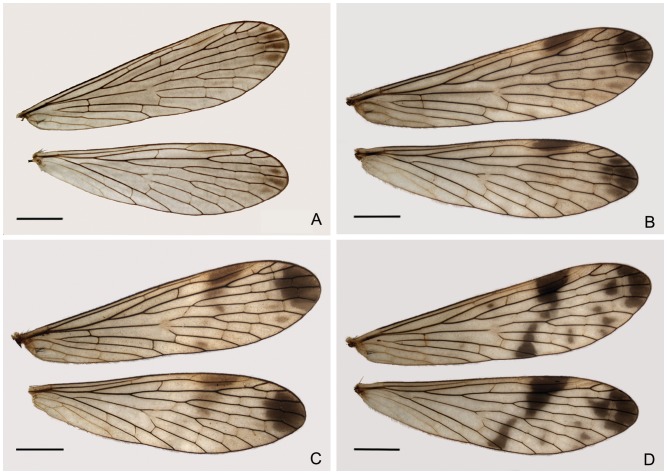
Variation of female wing markings of *P. obtusa*. Scale bars = 2 mm.

The female wings can be roughly categorized into four main types. Type I (28.3%) has dusky hyaline wings, without markings except for an indistinct grayish brown apical band and pterostigma (Fig. 7A). Type II wings (28.3%) bear a distinct small apical band and a pterostigmal band that is reduced to a large brown spot covering the pterostigma (Fig. 7B). The wings of type III (22.64%) bear a large distinct brown apical band and a light brown pterostigmal band that only reaches vein M_1_ (Fig. 7C). Type IV wings (9.43%) bear a distinct dark brown apical band, which appears broken as a prominent band and several small spots, and the prominent dark brown pterostigmal band with the basal branch complete and the distal branch reduced to a small spot (Fig. 7D).

#### Variation of the female genitalia

**Figure 8:**
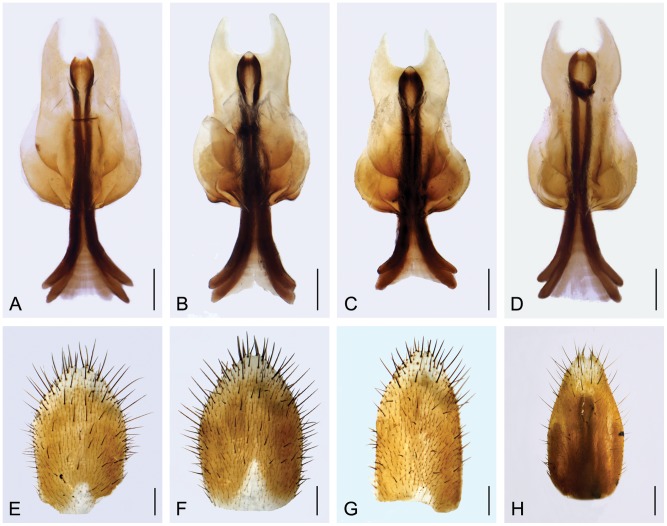
Variation of the female genitalia of *P. obtusa*, ventral views. (**A**)–(**D**) Genital plates. (**E**)–(**G**) Subgenital plates. (**H**) Subgenital plate with part of the genital plate adhering dorsally. Scale bars = 0.2 mm.

The genital plate varies mainly in the degree of constriction of the main plate, the emargination shape of the posterior arms, and the size of the ventral and dorsal basal plates (Fig. 8). For example, four types as shown in Figure 8 can be recognized mainly based on the constriction degree of the main plate, with their frequencies as 45.28%, 5.66%, 32.08%, and 16.98%, respectively (Figs. 8A−D). The subgenital plate varies from narrow to broad, with the apical V-shaped emargination randomly distinct or indistinct (Figs. 8E−H).

#### Type locality and habitat

At its type locality Taibaishan (34°04′N, 107°42′E) in the Qinling Mountains, *P. obtusa* is mainly distributed vertically from 1200 to 2300 m, abundant from the Shangbaiyun Temple (1780 m) via the Luotuoshi Temple (2050 m) to the Dadian Temple (2270 m). The adults emerge from the middle of May to early August and prefer resting on the leaves of bushes or grasses in moist habitats.

## Discussion

Previous taxonomic studies of Panorpidae have often been impaired by the variability of characters used [11]. The existence of intricate intraspecific or interspecific variation in Panorpidae causes the species delimitation difficult and often leads to synonym problems. This situation is especially remarkable in the largest genus *Panorpa*. Sibling species of *Panorpa* usually occupy only slightly different niches or even common habitat and are separated by biological means, despite exhibiting a considerable degree of morphological overlap [12]. *Panorpa debilis* Westwood, 1846, for example, has *P. canadensis* Banks, 1895 as its synonym and consists of five forms, which differ in characteristics of the aedeagus and distributions, suggesting the possibility of cryptic species [12]. The European *Panorpa alpina* Rambur, 1842 exhibits wide morphological variation in the male and female genitalia, and has been shown to have five synonyms [13]. On the basis of color and morphological variation of the head, thorax, abdomen, and genitalia in the *Panorpa cognata*-complex, 11 original species names have been evaluated and combined as four species with seven synonyms [10]. The Japanese scorpionfly *Panorpa japonica* Thunberg, 1784 has been proved to have 14 synonyms [14]–[16].

In this study, we found that both the males and females of *P. obtusa* collected from the type locality express intricate continuous variation in their phenotypes, especially in the wing markings and genitalia. All the described characters of *P. leei* can be found within the individual variation range of *P. obtusa*. In other words, *P. leei* is totally comprised of one of the morph types of *P. obtusa*. In combination with their identical type localities and overlapping morphological variation, it is reasonable to treat *P. leei* as a junior subjective synonym of *P. obtusa*.

In the face of the ‘taxonomy crisis’ and ‘biodiversity crisis’, contemporary taxonomists have to face a huge challenge to accelerate the rate of discovering and describing new species [17], [18]. However, since the past traditional taxonomic practice of insects was often conducted not only with poor optical equipment but also the lack of sufficient consideration of intraspecific variation of morphological characters, taxonomists have to cope with the burdensome legacy of past work to revise ubiquitous synonyms [18]. Synonyms are annoying but unavoidable by-products of taxonomic practice, greatly imperiling the estimation of alpha-biodiversity, especially in insect taxonomy.

Structural variation as a major source of characters and character states in traditional taxonomy [19], [20] is frequently excluded or ignored by morphological taxonomists, especially when the characters show any or “too much” variation [21]. Because morphological descriptions are difficult to standardize and intraspecific variation is prevalent in many insect groups, it is often difficult to determine whether character variation is intraspeific or interspecific [22]. Although many modern approaches have been used in current taxonomy [3], [23], [24], a large number of species will very likely continue to be routinely described and published based on morphological characters alone. Because synonyms caused by ignorance of the described entity can be dramatically reduced or even eradicated in the age of the Internet, modern taxonomists should pay more attention to widespread intraspecific variation and take steps to avoid new synonym generation as far as possible. One of the feasible ways may lie in new species descriptions that are based on a series of specimens. In other words, publishing new species descriptions based only on a single type specimen should be avoided as far as possible.

## Supporting Information

Table S1 **Frequency of morph types in** ***Panorpa obtusa*** **sampled.**
(PDF)

Table S2 **Measurements of characters in** ***Panorpa obtusa*** **sampled.**
(PDF)
