# Supplementary material for: Morphological Variation of the Scorpionfly Panorpa obtusa Cheng (Mecoptera: Panorpidae) with a New Synonym
Source: PLoS One. 2014 Sep 24;9(9):e108545. doi: 10.1371/journal.pone.0108545 (PMC4177224; doi:10.1371/journal.pone.0108545)
Supplement: Table S1 — Frequency of morph types in Panorpa obtusa sampled. (PDF) [file pone.0108545.s001.pdf]

Table S1. Frequency of morph types in *Panorpa obtusa* sampled

Characters		Total	Morphs	Individual number	Percentage (%)
Male	Wing	93	I	44	47.31
			II	24	25.81
			III	22	23.66
			IV	3	3.23
	Hapovalve	85	I	46	54.12
			II	36	42.35
			III	3	3.53
	Epandrium	93	I	59	63.44
			II	31	36.56
			III	3	3.23
	Paramere	90	I	20	22.22
			II	22	24.44
			III	28	31.11
			IV	20	22.22
Female	Wing	53	I	15	28.3
			II	15	28.3
			III	12	22.64
			IV	11	20.75
	Genital plate	53	I	24	45.28
			II	3	5.66
			III	17	32.08
			IV	9	16.98
	Subgenital Plate	33	I	16	48.48
			II	17	51.52
