# Supplementary material for: Morphological Variation of the Scorpionfly Panorpa obtusa Cheng (Mecoptera: Panorpidae) with a New Synonym
Source: PLoS One. 2014 Sep 24;9(9):e108545. doi: 10.1371/journal.pone.0108545 (PMC4177224; doi:10.1371/journal.pone.0108545)
Supplement: Table S2 — Measurements of characters in Panorpa obtusa sampled. (PDF) [file pone.0108545.s002.pdf]

Table S2. Measurements of characters in *Panorpa obtusa* sampled

<table>
<tr><th colspan="2">Characters</th><th>Individual number</th><th>Length (mean ± SD, mm)</th><th>Width (mean ± SD, mm)</th><th>Variation range (mm)</th></tr>
<tr><td rowspan="7">Male</td><td>Forewing</td><td>93</td><td>12.9 ± 0.50</td><td>3.50 ± 0.16</td><td>–</td></tr>
<tr><td>Hindwing</td><td>93</td><td>12.0 ± 0.50</td><td>3.40 ± 0.15</td><td>–</td></tr>
<tr><td rowspan="3">Hypandrium (Hypovalve)</td><td>31</td><td rowspan="3">1.30 ± 0.09</td><td>–</td><td>1.10 − 1.23</td></tr>
<tr><td>15</td><td>–</td><td>1.24 − 1.37</td></tr>
<tr><td>47</td><td>–</td><td>1.38 − 1.46</td></tr>
<tr><td>Gonocoxite</td><td>93</td><td>1.77 ± 0.08</td><td>–</td><td>1.56 − 1.86</td></tr>
<tr><td>Gonostylus</td><td>93</td><td>1.04 ± 0.03</td><td>–</td><td>0.96 − 1.13</td></tr>
<tr><td rowspan="2">Female</td><td>Forewing</td><td>53</td><td>13.1 ± 0.50</td><td>3.70 ± 0.20</td><td>–</td></tr>
<tr><td>Hindwing</td><td>53</td><td>10.9 ± 0.40</td><td>3.60 ± 0.20</td><td>–</td></tr>
</table>
